# An action research protocol to strengthen system-wide inter-professional learning and practice [LP0775514]

**DOI:** 10.1186/1472-6963-7-144

**Published:** 2007-09-13

**Authors:** Jeffrey Braithwaite, Johanna I Westbrook, A Ruth Foxwell, Rosalie Boyce, Timothy Devinney, Marc Budge, Karen Murphy, Mary-Ann Ryall, Jenny Beutel, Rebecca Vanderheide, Elizabeth Renton, Joanne Travaglia, Judy Stone, Amanda Barnard, David Greenfield, Angus Corbett, Peter Nugus, Robyn Clay-Williams

**Affiliations:** 1 Centre for Clinical Governance Research, Faculty of Medicine, University of New South Wales, 10 Arthur St, Kensington, NSW 2052, Australia; 2 School of Public Health and Community Medicine, Faculty of Medicine, University of New South Wales, Samuels Building, Kensington, NSW 2052, Australia; 3 Health Informatics Research & Evaluation Unit, Faculty of Health Sciences, The University of Sydney, 75 East St Lidcombe, NSW 1825, Australia; 4 University of Canberra, ACT 2601, Australia; 5 School of Health & Rehabilitation Sciences, The University of Queensland, St Lucia, Queensland 4072, Australia; 6 Australian Graduate School of Management, University of New South Wales, Sydney NSW 2052, Australia; 7 ANU Medical School, The Australian National University, C/- The Canberra Hospital, PO Box 11, ACT 2606, Australia; 8 ACT Health, Allied Health Adviser's Office, Level 2, 11 Moore Street, Canberra City, ACT, Australia; 9 ACT Health, c/- 11 Moore St Canberra City, ACT, Australia; 10 Department of Health, CitiCentre Building, 11 Hindmarsh Square, Adelaide South Australia, 5000, Australia; 11 School of Health Sciences – Nursing, University of Canberra, Canberra, ACT 2601, Australia; 12 The Canberra Hospital, PO Box 11, ACT 2606, Australia; 13 Faculty of Law, University of New South Wales, Sydney, NSW 2052, Australia

## Abstract

**Background:**

Inter-professional learning (IPL) and inter-professional practice (IPP) are thought to be critical determinants of effective care, improved quality and safety and enhanced provider morale, yet few empirical studies have demonstrated this. Whole-of-system research is even less prevalent. We aim to provide a four year, multi-method, multi-collaborator action research program of IPL and IPP in defined, bounded health and education systems located in the Australian Capital Territory (ACT). The project is funded by the Australian Research Council under its industry Linkage Program.

**Methods/Design:**

The program of research will examine in four inter-related, prospective studies, progress with IPL and IPP across tertiary education providers, professional education, regulatory and registration bodies, the ACT health system's streams of care activities and teams, units and wards of the provider facilities of the ACT health system. One key focus will be on push-pull mechanisms, ie, how the education sector creates student-enabled IPP and the health sector demands IPL-oriented practitioners. The studies will examine four research aims and meet 20 research project objectives in a comprehensive evaluation of ongoing progress with IPL and IPP.

**Discussion:**

IPP and IPL are said to be cornerstones of health system reforms. We will measure progress across an entire health system and the clinical and professional education systems that feed into it. The value of multi-methods, partnership research and a bi-directional push-pull model of IPL and IPP will be tested. Widespread dissemination of results to practitioners, policymakers, managers and researchers will be a key project goal.

## Background

### Introduction

Industries as diverse as health [1,2], aviation [3], manufacturing [4], finance [5], education [6] and the military [7] have identified that collaborative learning and team-based practices are key drivers of performance improvement, safer organisations and systems renewal. Yet there is limited research which demonstrates convincing models of inter-professional learning (IPL) (also titled inter-professional education, IPE) and inter-professional practice (IPP) that successfully achieve these outcomes, particularly across whole health systems. This is a substantial issue requiring attention.

This project's broad goal is to use IPL as the basis for improving IPP, which in turn is thought to lead to enhanced safety and quality of care for patients, and morale and outcomes for patients, staff and students [8,9], across an entire health system. This means the project stands at the intersection of three industries – tertiary education, professionally-based education, and the health system, and it spans both the public and private heath sectors. Specifically, the project will achieve its overarching goal through an Australian Research Council (ARC) funded action research project to strengthen IPL and IPP across the Australian Capital Territory (ACT) health and tertiary and professional education systems.

By action research we mean disseminating findings to participants and other stakeholders, encouraging bi-directional feedback and enabling reflection to stimulate productive change and improvement in a participatory environment. The scientific innovation we aim to realise is to enhance systems-wide teamwork and collaboration through the application and testing of a new model of IPL and IPP in order to bring about profound culture change in the way health professionals work together to deliver services. The project contributes the largest, most comprehensive effort to achieve this yet attempted. We define IPL as "a collaborative, interdisciplinary education and learning process designed to produce effective, multidisciplinary patient centred care" [10] and IPP is the enactment of competencies required to attain this [11]. IPL involves educating clinical professional staff (doctors, nurses and midwives, and allied health practitioners) in multidisciplinary approaches with the aim of encouraging IPP – ie, greater levels of teamwork, collaboration, knowledge-sharing and problem-solving in health settings.

### The significance of IPL and IPP

Why are IPL and IPP important; how will they contribute to improvements? Why is this project significant? Previous studies, reviews and commentaries have suggested that IPL can lead to collaborative IPP amongst clinicians and clinical groups [12,13]. This, in turn, is believed to contribute to safer and higher quality services to patients, and improved morale for staff and students [14,15]. The literature suggests that patient care (or, outside of health care, services to customers more generally) will be improved by stronger practitioner relationships, teamwork and inter-professional communication.

Summarising this claim, we have argued that "IPL is centrally concerned with improving the way people work together so that clinicians can grow professionally, learn from others, provide support to colleagues and improve the quality of care to patients" [16]. The putative benefits of IPL and IPP have been well documented: enhanced communication and trust amongst clinical groups [17], collaborative skills [18], reductions in between-professional rivalries [19], and better professional relationships [20]. IPL is held to build team approaches [21], and lead to more creative, integrated services [22]. IPL is said to help students understand how to contribute effectively with other disciplines [23]. Establishing common educational curricula across health professional groups will logically help create common philosophies, languages, perspectives and values [24] and enable skills transfers across the professional silos that prevail today [25].

However, there have been criticisms to balance these optimistic assessments. Progress to date has been slow and uneven. Some tertiary education providers have been uncomfortable about IPL. Existing professionally-based educational structures and practices facilitate specialisation, and maintain medical, nursing and allied health traditions and unique contributions, albeit at the expense of teamwork [26]. Others have argued that IPL and IPP can be riven with unclear philosophies, replete with muddled thinking and multiple objectives [27], and they might often have more theoretical potential than actual importance [28]. The sharpest criticism comes from those who argue that IPL and IPP advocates have failed to provide strong evidence for their claims. The data supporting the proposition that IPL influences IPP to create sustained systems change is weak and diffuse, and the evidence largely comprises non-transferable case study, survey and other limited data [29,30]. There is no level 1, randomised study showing convincingly that IPL has worked; but neither is there strong level 1 evidence showing that it does not [31,32]. Deeper, more extensive evaluation efforts are therefore required and action research demonstrations of IPL and IPP are needed. Ideally, we would study an entire health system in a project that involved multi-methods and multiple levels; it is time to do so.

The action research project we propose addresses this crucial problem. It is hard to overestimate its importance. Health is an exemplar industry requiring effective teamwork: whenever things go wrong in health care, reports [33], enquiries [34] and studies [35] show that a predetermining factor is that patient care is delivered in a fragmented, isolated way, with health-care professionals having failed to collaborate effectively. Safety is compromised and quality suffers in such circumstances [36]. Internationally, the rate of adverse events – incidents which harm patients, caused by the health care system itself – is estimated to occur in 10% of all admissions [37]. It is well established that in the order of 18,000 Australian patients die and 50,000 patients are disabled annually and major causes include poor communication and lack of teamwork as well as tribal, non-collaborative structures [38]. IPL and IPP are thus argued to be crucial underlying determinants of safer acute care models and improved quality of services.

### The case for the project

There is a growing understanding amongst policymakers, educators and clinicians that IPL's contribution, and the enactment of IPP, are important but as yet unrealised. For example, the Health Workforce Advisory Committee of the New Zealand Government has argued: *"Health practitioners must learn to work in teams whose aim is to provide safe, high-quality, integrated and well-managed care that makes best use, in the widest sense, of all the resources a community has to commit to health .... To achieve this will require changes to the way health practitioners are trained and deployed, and to the way they work" *[39]. The Institute of Medicine (IOM) in the United States of America put it this way: *"Clinical education simply has not kept pace with or been responsive enough to shifting patient demographics and desires, changing health system expectations, evolving practice requirements and staffing arrangements, new information, a focus on improving quality, or new technologies .... Once in practice, health professionals are asked to work in interdisciplinary teams, often to support those with chronic conditions, yet they are not educated together or trained in team-based skills" *[40]. Canada's Commission on the Future of Health Care agrees: *"If health care providers are expected to work together and share expertise in a team environment, it makes sense that their education and training should prepare them for this type of working arrangement" *[41]. The National Health Service (NHS) in the United Kingdom has expressed a confirming view: *"All health professionals should expect their education and training to include common learning with other professions" *[42].

Quite simply, however, regardless of the favourable admonitions, no one has put a research team in the field under the right conditions (with receptive research partners, a health and related education system with a strong readiness to engage and a motivated and skilled workforce) to do this work, despite the widespread international agreement about the imperative for systems-wide IPL and IPP. We conducted a literature review on IPL and IPP [10] in preparation for the partnership's work together in 2005 and to design this project. This has helped position ACT Health and the partners and has laid the platform for IPL and IPP across the Territory. We uncovered 37,812 references. We subjected these primary references to a content analysis using Leximancer, a software analysis tool to create a conceptual map of IPL and IPP. We conducted a secondary refining process and excluded non-substantial, atheoretical, non-empirical and less relevant articles. The usable literature set comprised 3,765 references which were reviewed by two independent researchers, sorted into categories and further synthesised. We found no previous study of the kind we are describing here, internationally or locally. In as rigorous a way as is possible therefore we will be advancing the knowledge base in this area.

## Methods/Design

### Collaborators

**Figure 1:**
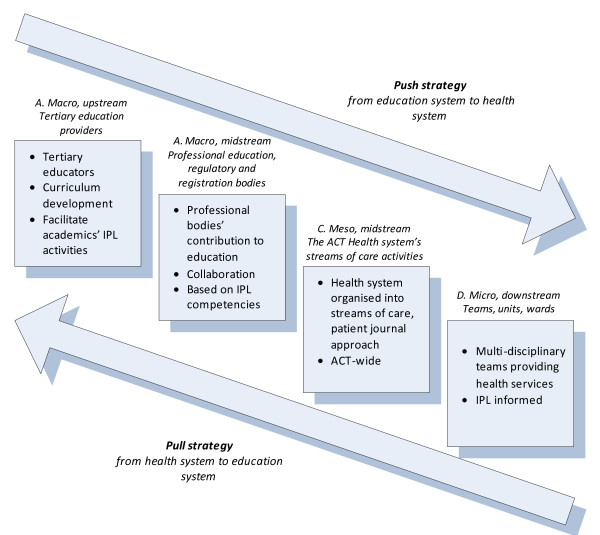
Four areas of focus for the IPL project.

We aim to produce an action research demonstration and conduct an extensive formative and summative evaluation of IPL and IPP through a project in which we work collaboratively in a team-based, researcher-industry partnership to assess four components of IPL (Figure 1) over a four year enactment period. The Centre for Clinical Governance Research (CCGR) at University of New South Wales (UNSW) provides research leadership to the project. ACT Health is a public health system of 5,683 people (4,869 full-time equivalent staff, FTE) at February 2006, providing 417,186 episodes of inpatient care in 2004–2005 as well as 427,685 community health and 226,908 community mental health occasions of service. It has a budget of $671.3 million. It is embarking in concert with project partners on a large-scale, comprehensive, longitudinal process of attempting to influence and institutionalise IPL in four domains: first, through major tertiary health sector education providers (eg, Australian Catholic University (ACU), Australian National University (ANU), Canberra Institute of Technology (CIT) and University of Canberra (UC); second, amongst professional education, regulatory and registration bodies (eg, Australian Physiotherapy Association (APA), Royal College of Nursing, Australia (RCNA), Royal Australian College of Physicians (RACP), Royal Australasian College of Surgeons (RACS), Royal Australian College of General Practitioners (RACGP), the regional General Practice (GP) registrar training group – CoastCityCountry Training, the Council of Pharmacy Registering Authorities (CPRA), and various ACT Registration Boards); third, via the streams of care through which ACT Health provides Territory-wide services (eg Community Health Services, Mental Health ACT, Capital Region Cancer Services, Aged Care & Rehabilitation Service); and fourth, in teams, wards and departments that provide local services (eg, surgical, medical, emergency units).

### Research design

We have designed four interrelated prospective studies to meet our aims and objectives. Our design conducts this research project with four components in a sustainable study: macro, upstream, involving tertiary education providers; macro, midstream, involving professional education, regulatory and registration bodies; meso, midstream, involving ACT Health's streams of care activities; and micro, downstream, involving teams, units and wards. As Figure 1 schematically argues, an action research project in the way we have conceptualised, depending on the strength of the enabling forces, can provide the push and pull needed for take up and institutionalisation of IPL and IPP.

**Figure 2:**
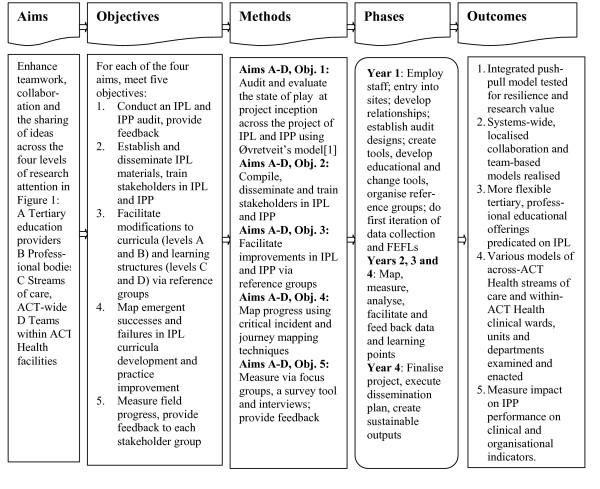
Research approach.

While it is true to say that the case has been advanced since the 1960s that IPL and IPP are important, recent work including from the IOM in *Health professions education: a bridge to quality *[40], the report of the Pew Commission [43], the Centre for Inter-professional Education and Research [44], the Higher Education Academy Health Services Practice Network [45] and various other commentators have made strong arguments underpinning the kind of research design proposed here. But research into IPL's and IPP's effectiveness has never been done before in this way, across an entire health system. An action research approach with a motivated partner organisation has much to offer, including potential for breakthrough performance gains in practice, rather than in theory, in isolated case studies, or in the laboratory. Conducting a project at four levels is challenging but is what is needed to achieve the much-vaunted but rarely realised workforce-enabled systems change. This is necessary for effective health reform and to provide a demonstration for other sectors which are in need of enhanced teamwork. Focusing on a push-pull model, and using improvement cycles based on formative evaluation feedback loops (FEFLs, see Figure 2), have much to recommend them. Providing bi-directional feedback loops is a bespoke strategy based on our collective expertise in conducting research and evaluation projects [46,47]. This is research which is conceptually advanced and original, as we are testing a theoretical model never before used; our four-level, push-pull model has been purpose-designed for the task at hand.

### Aims and objectives

Few studies illuminate research progress at four levels of a complex system, and even fewer harness the pressure of a push-pull model to try to invoke sustainable change. We are striving for an integrated strategy from education through to practice, and practice through to education, and aim to create culture change across all four levels. In systems dynamics terms this, if successful, becomes self-reinforcing. The project has four specific research aims and it will attempt to address these, and 20 research objectives, over four years; see Figure 2, *Research approach*.

The aims are to enhance teamwork, collaboration and the sharing of ideas, knowledge and practice amongst:

A. Educational facilities and learners in tertiary educational programs associated with ACT Health

B. Professional education, and regulatory and registration bodies providing education to their members, associated with ACT Health

C. Clinicians in ACT Health's streams of care

D. Teams, wards and units throughout ACT Health.

Each of the aims has five project objectives. These are as follows.

1. At project inception conduct an IPL and IPP audit of each stakeholder group in A-D above

2. Establish a repository of education-oriented IPL materials, disseminate these to partners and others involved in the project, and train stakeholder groups A-D in IPL and IPP

3. Facilitate modifications to tertiary and professional educational curricula (stakeholder groups A and B) and learning structures in streams of care and team-based units (stakeholder groups C and D) through various reference groups for each of A-D at each level of the project to guide and promote curricula and practice change

4. Map emergent successes and failures in IPL and IPP progress amongst stakeholder groups A-D against curricula developments and practice changes at six months and thereafter annually using critical incident and journey mapping techniques [48] and provide feedback to participants using FEFL strategies to encourage further IPL and IPP improvements

5. Measure progress via focus groups, questionnaire survey administration and key stakeholder interviews at predetermined marker times – at nine months, one year nine months, two years nine months and three years nine months of the project and provide feedback to stakeholder groups A-D using FEFLs to encourage further IPL and IPP improvements.

The project will examine IPL and IPP processes in the Territory at the project's inception and as it unfolds, and gather baseline and ongoing data to measure project progress. It will enhance take-up through the FEFL strategy. These data will provide an evidence base for ongoing project enhancements.

### Research plan

**Figure 3:**
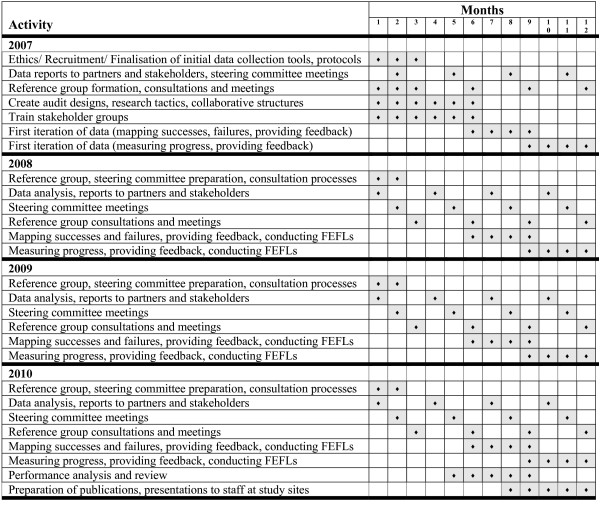
Detailed research plan and process.

Figure 3 shows how the project will play out in a detailed research plan and process. This builds on the collaborative partnership, action research ideals of the research design.

## Discussion

### IPL and IPP as cornerstones of health care reform

IPL and IPP are increasingly seen as building blocks to progress in health care. The ageing workforce [49], projected shortages of staff [50], problematic morale because of isolated groups and autonomy [51] and disaffection with existing fragmented health services [52] are seen by the Australian Productivity Commission [53], amongst others, to be in need of attention. We have worked throughout 2005 and 2006 in a partnership involving jointly-conducted meetings, reference group sessions, workshops, seminars, consultancy processes and network-building underpinned by an extended literature review [10] and discussion documents [11, 54, 55, 56] which have been circulated throughout ACT Health. We designed an IPL framework [16] and implementation plan [57] to underpin IPL's institutionalisation throughout the ACT health and associated education systems. The main tertiary health education providers at ACU, ANU, CIT and UC are committed to this project and have confirmed their willingness and capacity to engender flexibility in educational offerings. We have secured high-level endorsement for this project and recognition of its importance from the Minister for Health in the ACT Government, the Director-General of Health for the ACT and the Commissioner who led the recent Productivity Commission inquiry which culminated in the report *Australia's health workforce *[53], a major reform document. Additionally, the project drew on the other advanced international work in this field [58].

### The benefits of multi-method, partnership research

Projects of this type are essentially measuring progress formatively, invoking capacity-building measures and attempting to build substantial research assets for the future. The partners enabling these research aims to be met have co-designed the ACT Health's IPL initiative and the action research project specified here. Success is dependent on strong partnerships and robust relationships.

ACT Health is a highly motivated partner and has been an instrumental participant in the development of the study. Staff across the ACT have participated in a reference group last year [n = 65], and consultations have been conducted about this project with approximately 200 staff members. ACT Health and the ARC project investigators and other partners have facilitated discussions with a wide range of stakeholder groups at the four levels identified in Figure 1. The partners have also conducted in late 2006 an Australia-wide inter-jurisdictional workshop for educational providers, regulators, registration bodies and practitioners. Senior policy, managerial and clinical staff have been involved in the detailed research design developments and several staff members are beginning the process of enrolling in a PhD in the topic area. Many ACT Health staff are keen to be involved in the studies and are willing to commit time as an adjunct to their usual workload. They have a range of experiences and expertise that will provide critical knowledge to the studies.

ACT Health's *Health action plan*, its primary document setting strategic directions, advocates a considered approach to health status improvement for the Territory's residents, calling, *inter alia*, for *healthy systems – building a sustainable workforce*, *integrating hospital and community health services *and *enhancing the quality of health and community services*. Although IPL and IPP underpin the various components of the *Health action plan's *proposals, nevertheless it contributes particularly strongly to these three aspects.

The project is valuable to ACT Health in numerous ways, as the foregoing articulates. If we can achieve our aims and realise this project's contributions we will have enabled major team-based, IPP-oriented improvements. Even if we make only modest or uneven progress we stand to create a platform for improvement, underpinning future initiatives designed to create sustained systems change. These are goals worth pursuing.

In essence, the research aims and objectives posed require a multi-method [59], multi-level approach [60] which incorporates both qualitative and quantitative data [61]. The research program investigates performance in terms of empirical data, what people say occurs (focus groups, interviews and survey questionnaires) and observations of what occurs (critical incident mapping, reference group experiences and field observations). A wide range of evaluation techniques will thus be applied in meeting the study aims and objectives. This is thus innovative research of high strategic value.

### Closing the loop by communicating results

We have designed multiple strategies by which to communicate results. Too often formative results of large-scale research projects lie dormant or are unpublished. First, we are in the process of establishing a website to facilitate dissemination of our findings and lessons learnt, targeted to our partners and stakeholders as well as other interested parties nationally and internationally. Second, we are keen to diffuse results. We will do this via conference, symposia and workshop presentations and academic journals. Third, we are beginning to target multiple external stakeholders who are interested in the findings and their implications. To do this we intend designing useful summaries for the benefit of interested parties. Whenever we have done this previously we have been struck by the way this is warmly received. Fourth, we are motivated to share our experiences with the multi-method research model at the heart of this project. In testing a publicly-funded multi-method model and investigating how this works there is a moral imperative to share this with other researchers and practitioners.

Most important is the research translational competencies we intend applying to this project. We seek to go beyond communications of results; this project itself is based on an action research model but collectively we are committed to translational research. We have involved a very wide range of partners and stakeholders and secured their expertise, engaged them and sought their active involvement to develop the research protocol. We plan to conduct media interviews and public lectures about how our results fit into health sector reform as they emerge, and how to improve the way in which professionals work together. We are also arranging to give lectures to senior high school students to influence their career choices and prepare them for inter-professional learning and practice when they go into the workforce.

## Conclusion

Systems-wide, empirical evidence about IPL and IPP is sparse. We seek to redress this deficit in part via this large-scale project. A research design predicated on multi-method, multi-disciplinary, multi-level collaborative principles is one that is likely to reflect the complexity of the issues to be investigated. In disseminating this research protocol through open access we aim to generate debate about the possibilities of success, to generate feedback from colleagues, and to create a dialogue with other interested researchers in this area.

## Abbreviations

ACT, Australian Capital Territory; ACT Health, the Australian Capital Territory health service provider; ACU, Australian Catholic University; AGSM, Australian Graduate School of Management; ANU, Australian National University; APA, Australian Physiotherapy Association; ARC, Australian Research Council; CCGR, Centre for Clinical Governance Research at University of New South Wales; CIT, Canberra Institute of Technology; CPRA, Council of Pharmacy Registering Authorities; FEFL, Formative Evaluation Feedback Loop; FTE, Full-Time Equivalent; GP, General Practice; IOM, Institute of Medicine, United States of America; IPE, Inter-professional education; IPL, Inter-professional learning; IPP, Inter-professional practice; NHS, National Health Service; RACGP, Royal Australian College of General Practitioners; RACP, Royal Australasian College of Physicians; RACS, Royal Australasian College of Surgeons; RCNA, Royal College of Nursing, Australia; UC, University of Canberra

## Competing interests

This research is supported under the Australian Research Council's Linkage Projects funding scheme [project number LP0775514] which brings together industry partners [represented by KM] and academics [led by JB] to undertake partnership research. While no-one reports any financial or non-financial competing interests, these kinds of research partnerships require the development of appropriate safeguards, including a clear understanding of roles, responsibilities and the arm's length nature of academic researchers. There is an acceptance of the collaborators that results may differ from their expectations and be disadvantageous to their interests, but nevertheless publication will be pursued.

## Authors' contributions

JB, JW, ARF, RB, TD and MB are chief investigators on the grant and made substantial contributions to the conception and design of the project and this manuscript. KM and M-AR are industry partner investigators, as was JBe at the time of the development of the protocol. RV, ER, JT, JS, AB, DG, AC, PN and RC-W contributed at various times to the project's conceptualisation, as members of one of the steering committees or are involved in one or more components of the research studies. All authors contributed to this project and the paper and read, and approved, the final manuscript.

## Pre-publication history

The pre-publication history for this paper can be accessed here:


